# Phylogeography and Coevolution of Bamboo Mosaic Virus and Its Associated Satellite RNA

**DOI:** 10.3389/fmicb.2017.00886

**Published:** 2017-05-23

**Authors:** Ing-Nang Wang, Wen-Bin Yeh, Na-Sheng Lin

**Affiliations:** ^1^Department of Biological Sciences, University at Albany, AlbanyNY, United States; ^2^Department of Entomology, National Chung Hsin UniversityTaichung, Taiwan; ^3^Institute of Plant and Microbial Biology, Academia SinicaTaipei, Taiwan

**Keywords:** BaMV, satBaMV, phylogeography, cophylogeny, evolution

## Abstract

*Bamboo mosaic virus* (BaMV), a plant potexvirus, has been found only in infected bamboo species. It is frequently associated with a large, linear single-stranded satellite RNA (satBaMV) that encodes a non-structural protein. Decades of collecting across a wide geographic area in Asia have accumulated a sizable number of BaMV and satBaMV isolates. In this study, we reconstructed the BaMV phylogeny and satBaMV phylogeny with partial coat protein gene sequences and partial genomic sequences, respectively. The evolutionary relationships allowed us to infer the phylogeography of BaMV and satBaMV on the Asian continent and its outlying islands. The BaMV phylogeny suggests that the BaMV isolates from Taiwan, unsurprisingly, are most likely derived from China. Interestingly, the newly available satBaMV isolates from China were found to be most closely related to the previously established Clade III, which is found in India. The general pattern of clustering along the China/India and Taiwan divide led us to hypothesize that the Taiwan Strait has been a physical barrier to gene flow in the past evolutionary history of both BaMV and satBaMV. Lastly, cophylogeny analyses revealed a complex association pattern between BaMV and satBaMV isolates from China. In general, closely related BaMV sequences tend to carry closely related satBaMV sequences as well; but instances of mismatching with distantly related satBaMV isolates were also found. We hypothesize plausible scenarios of infection and superinfection of bamboo hosts that may be responsible for the observed association pattern. However, a more systematic sampling throughout the geographic distribution of various bamboo species is needed to unambiguously establish the origin, movement, and evolution of BaMV and satBaMV.

## Introduction

Population genetic surveys and phylogenetic reconstructions of plant viruses and their associated satellites offer us invaluable insights into not only the observed patterns and inferred processes of plant virus evolution (García-Arenal et al., 2003) but also the ecological interactions that may contribute to long-distance dispersal and emergence of new diseases (Fargette et al., 2006). Ever since the enigmatic RNA 5 was confirmed to be a satellite RNA (satRNA) associated with the *Cucumber mosaic virus* (CMV) (Kaper et al., 1976), this model system has become the most widely studied combination of helper virus and satRNA. Recent works span their basic biology (Palukaitis et al., 1992; Roossinck et al., 1992; García-Arenal and Palukaitis, 1999; Palukaitis and García-Arenal, 2003; Jacquemond, 2012), their ecology (Gallitelli, 2000) and evolution (Roossinck, 2001, 2002). Especially interesting is a series of field surveys and subsequent experimental studies that investigate a multi-year epidemic of CMV and its associated satRNAs (satCMV) in Eastern Spain (Fraile and García-Arenal, 1991; Jordá et al., 1992; Aranda et al., 1993; Alonso-Prados et al., 1998; Escriu et al., 2000a,b; Betancourt et al., 2011). A multi-year epidemic ravaged by CMV and satCMV in Italy yielded a similar pattern of genetic variation in the field (Grieco et al., 1997). Various phylogenetic and phylogeographic studies of plant viruses and their associated satellites in nature have revealed clustering of isolates based on symptoms, plant hosts, and/or geographic origins (Grieco et al., 1997; Cabrera et al., 2000; Abubakar et al., 2003; Pinel et al., 2003; Fargette et al., 2004; Tomitaka and Ohshima, 2006; Olarte Castillo et al., 2011; Venkataravanappa et al., 2011; Cuevas et al., 2012a,b). However, it is to be noted that most of these studies are with viruses that adopt the acute lifestyle (Roossinck, 2010), infecting plant hosts that are predominantly annual crops.

Bamboos are a group of evergreen perennial grasses belonging to the grass family Poaceae (Kelchner and Bamboo Phylogeny Group, 2013; Clark et al., 2015). There are approximately 1,400 to 1,500 bamboo species, distributed worldwide, except Europe and Antarctica (Kelchner and Bamboo Phylogeny Group, 2013; Clark et al., 2015). Of the two main bamboo types, the most economically, ecological, and culturally important, and also what we are most familiar with, is the woody bamboos (as opposed to the herbaceous bamboos). Of the reported bamboo diseases, only two are caused by viruses (Su and Wang, 2015). *Bamboo mosaic virus* (BaMV) was first recognized in Brazil in 1974 in two bamboo species, *Bambusa multiplex* and *B. vulgaris* (Lin et al., 1977). Besides South America, BaMV has subsequently been reported in various parts of the world, including North America, Asia, and Australia (Lin et al., 1992, 1993, 1995, 2015; Thomas and Dodman, 1999; Nelson and Borth, 2011). Curiously, no case of BaMV infection has been reported on the African continent, where many bamboo species are found (Clark et al., 2015). It should not come as a surprise if BaMV is eventually reported in Africa as well.

*Bamboo mosaic virus* belongs to the genus *Potexvirus* of the family *Alphaflexiviridae* (Adams et al., 2011). It is a flexuous rod of approximately 490 nm × 15 nm in size (Lin et al., 1977; DiMaio et al., 2015). Its single-stranded RNA genome is approximately 6,400 nucleotides long, encoding a polymerase, triple-gene block (proteins involved in cell-to-cell movement), and a coat protein (CP) (Lin et al., 1992, 1994). In the field, BaMV is frequently associated with a satellite RNA (satBaMV) (Lin and Hsu, 1994; Yeh et al., 2004; Wang et al., 2014). Although the genome of the type member, satBaMV-BSF4, is only 836 nucleotides long, it nevertheless belongs to a group of large linear single-stranded satellite RNAs (satRNAs) (Mayo, 1991; Briddon et al., 2012). However, unlike most other members of this satRNA group, all of which are all associated with helper viruses in the family *Secoviridae*, satBaMV is the only known example of satRNA associated with a member in the family *Alphaflexiviridae* (Mayo, 1991; Briddon et al., 2012). As is typical of this large satRNA group, satBaMV also encodes a non-structural protein, P20, which is involved in the systemic movement of satBaMV within the infected host (Lin et al., 1996; Palani et al., 2006, 2012; Chang et al., 2016). The P20 protein is the only known large satRNA-encoded protein not required for satRNA replication (Lin et al., 1996).

Since satBaMV depends on its helper virus for genome replication, cell-to-cell movement, and encapsidation, it can be viewed as a molecular parasite exploiting various vital functions of its host BaMV (Nee, 2000). That is, interaction between the help virus and its associated satRNA is typically seen as antagonistic, presumably via competition for accessing the viral encoded RNA-dependent RNA polymerase (RdRp) for replication (Wu and Kaper, 1995). For BaMV and satBaMV, the competition for RdRp is likely mediated through the untranslated 5′-end regions of their genomes (Chen et al., 2007, 2010, 2012). One of the consequences of such an interaction is manifested as the severity of the infected plant hosts’ symptoms (Collmer and Howell, 1992). BSF4 and BSL6 are two of the most frequently studied satBaMV isolates. Their disease symptoms, when coinfected with BaMV separately, represent the opposite ends of the spectrum, with BSF4 infection showing a severe mosaic symptom, while BSL6 infection a relatively mild one (Hsu et al., 1998). This apparent antagonistic interaction, albeit manifested with a wide range of symptom severity, is further complicated by other indirect interactions mediated through the defense mechanisms of the host plant (Hu et al., 2009). Consequently, the coevolutionary patterns may be host plant dependent. One possible consequence of long-term interaction is the evolution of helper virus specificity by the satRNA. For example, some large *Nepovirus*-associated satellite RNAs can be supported only by certain isolates or serotypes of their helper viruses (Murant and Mayo, 1982; Roossinck et al., 1992; Fritsch et al., 1993; Oncino et al., 1995). In the most extreme case, the specificity can simply be determined by the presence or absence of a single amino acid residue (Roossinck et al., 1997). But it should be noted that there is no solid empirical demonstration for the hypothesized arms race dynamics between isolates of BaMV and satBaMV, and consequently it is not clear to what extent these interactions are a driving force for sequence evolution.

In contrast to well-established plant virus systems, BaMV has not been subjected to phylogeographic investigation. However, limited studies with full-length genomic sequences do reveal a clustering pattern, and suggest that isolates from Taiwan are derived from China (Lin et al., 2016, 2017b). In comparison, we have previously reported a more extensive study on various satBaMV isolates sampled from parts of China, India, and Taiwan (Wang et al., 2014). Phylogenetic analysis uncovered three distinct and well-supported satBaMV clades that most likely have persisted for many decades, if not longer. Interestingly, there is no single characteristic, such as geographic origin or host bamboo species, defining these clades. For example, Clade I, exemplified by the type sequence BSF4, is composed of isolates from various BaMV-infected bamboo species on Taiwan and the Hainan Island of China and also, interestingly, one single sample from the United States. Isolates in Clade II, e.g., the type sequence BSL6, are almost exclusively found in Ma bamboo (*Dendrocalamus latiflorus* Munro) on Taiwan. Clade III is currently found only in India infecting *B. vulgaris*. As more samples from different geographic locations and host bamboo species are included in the analysis, it is not clear whether these three satBaMV clades will persist.

In this study, we take advantage of newly available BaMV and satBaMV sequences from China that were isolated from various bamboo species in three botanical garden settings (Lin et al., 2015, 2016, 2017a). We investigate how BaMV and satBaMV may have migrated across the Asian continent. We also hypothesize the mechanisms responsible for the observed cophylogeny pattern between BaMV and satBaMV.

## Materials and Methods

### Sequence Acquisition

We used sequence information of BaMV and its associated satBaMV from three sources: (1) novel sequences from China deposited at the GenBank (accession numbers KP233222 and KP256025-256071 for BaMV, and KP233223 and KP256110-KP256146 for satBaMV), (2) sequences from our previous studies (Yeh et al., 2004; Wang et al., 2014), and (3) BaMV CP gene sequences from Taiwan that are new to this study. In our previous study (Wang et al., 2014), a total of 568 sequences, most of which from Taiwan, were used to reconstruct the satBaMV tree. To facilitate phylogenetic reconstruction and to avoid over-representation of satBaMV sequences from Taiwan, we selected five sequences from each of the six groups (*B. oldhamii, B. vulgaris, D. latiflorus*, and *D. latiflorus* cv. Mei-nung from Taiwan; *B. ventricosa* from Hainan Island, China; and *B. vulgaris* from India) for this study. The goal of the selection is to have maximal diversity represented for each group. The selection is guided by within-group pairwise comparisons using MEGA7 v7.0.20 (Kumar et al., 2016). The new BaMV sequences from Taiwan were isolated in 1994–2000, from various infected bamboo species and locations (Yeh et al., 2004). BaMV virions from infected bamboo leaves were purified and the viral RNA extracted as described previously (Lin and Chen, 1991). Purified viral RNA was used as the template and oligonucleotides B81 (5′-ACGGGAGCTCT_20_-3′, underlined letters indicate the SacI site) as the primer for reverse transcription to synthesize the first strand cDNA. The primer pair of B81 and B43 (5′-CGACGTTGGAAATAATAATAAAC-3′, underlined letters indicate the BstXI site), which complements the 5′ flanking promotor region of the CP gene, were used to amplify the CP gene. The DNA amplicon was separated with 1% agarose gel, gel-purified, ligated into pGEM-T Easy cloning vector (Promega, Madison, WI, United States), and then transformed into *Escherichia coli* DH5α. The resulting plasmid, carrying the inserted sequence, was selected and sequenced. DNA sequencing was performed by ABI 377A Sequencer using the BigDye Terminator Cycle Sequencing Kit (Applied Biosystems, Foster City, CA, United States).

All new sequences are deposited at the GenBank. **Supplementary Material S1** lists all sequences used in this study, including isolate names, locations and dates of sampling, and GenBank accession numbers. **Supplementary Material S2** shows the map with the approximate sampling locations.

### Sequence Alignment

The recently available data from China are partial sequences of BaMV CP genes and partial genomic sequences of satBaMV. For sequence alignment, all other sequences were trimmed to the same lengths as those from China. To avoid confusion and to facilitate orientation, the BaMV-S and satBaMV-BSF4 genomic sequences, accession numbers AF018156 and AY205227, respectively, are used as references for positioning the nucleotide and protein sequences used in this study. For BaMV, the nucleotide sequences corresponding to the BaMV-S genomic sequence, nucleotides 5611–6138 (528 nucleotides; 72.4% of the CP gene sequence; encoding amino acid residues 39–213), are used. For satBaMV, the nucleotide sequences corresponding to the satBaMV-BSF4 genomic sequence, nucleotides 271–742 (472 nucleotides; 56.5% of the genomic sequence; encoding P20 amino acid residues 38–183), are used.

The most closely related sequences to BaMV CP and satBaMV P20 proteins are the CP proteins of the *Foxtail mosaic virus* (FoMV, GenBank accession number NC_001483) (Yamaji et al., 2001) and the *Panicum mosaic satellite virus* (SPMV, GenBank accession number NC_003847) (Liu and Lin, 1995), respectively. Therefore, these two sequences were used as outgroups to root the corresponding BaMV and satBaMV trees. Two pairs of full-length protein sequences, FoMV CP/BaMV-S CP and SPMV CP/satBaMV-BSF4 P20, were aligned separately using Expresso of the T-coffee online service^1^ (Notredame et al., 2000; Armougom et al., 2006; Di Tommaso et al., 2011). The partial BaMV and satBaMV nucleotide sequences were aligned using the online service, Clustal Omega^2^ (Sievers et al., 2011). The initial nucleotide sequence alignments were then manually adjusted using the protein sequence alignments as guides.

### Phylogenetic Reconstruction

We used both the Bayesian inference (BI) and maximum likelihood (ML) methods to infer the relationships among the aligned BaMV and satBaMV sequences. For each method, alignments with or without the outgroup sequences were also constructed. The substitution model “GTR+G+I,” selected by the automatic model selection function of the PhyML server version 3.0^3^ (Guindon et al., 2010), was used for all tree constructions, except for that of the BaMV ML tree with FoMV as the outgroup, for which the model “GTR+G” was used. For the BI method, MrBayes (Huelsenbeck and Ronquist, 2001), version 3.2 (Ronquist et al., 2012) running on Mac OS X version 10.12, was used. All reconstructions with MrBayes were run for 10,000,000 generations and had 25% burn-in. The convergence of the runs was assessed using the program Tracer v1.6 (Rambaut et al., 2014). For all runs, the ESS (effective sample size) values for the log likelihood LnL ranged from 1519 to 3196, and the PSRF (potential scale reduction factor) values were all close to 1.0. For the ML trees, we used the online PhyML server (see above). A bootstrap of 500 replicates was used to estimate branch support. We used the program TreeGraph 2 (Stöver and Müller, 2010) to collapse branches with low support values. For the BI trees, the criterion of branch collapsing is for the posterior probability <0.90, and for the ML trees the criterion is replicate number <375 (75%). The program FigTree, version 1.4.3 (Rambaut, 2012), was used to visualize and manipulate the trees, including the function of midpoint rooting.

### Cophylogeny Analysis

Both *Jane* version 4.0.1 (Conow et al., 2010) and *PAC*o version 1.1.r (Balbuena et al., 2013) were used for cophylogeny analysis. For analysis using *Jane*, we employed default costs of “cospeciation” = 0, “duplication” = 1, “duplication and host switch” = 2, “loss” = 1, and “failure to diverge” = 1. We found that the values for the Genetic Algorithm parameters have some effects on the estimated minimum cost. We conducted an initial exploration of the parameter space of the “number of generations” and “population size” by varying their values from the default of 100 to a range of 30 to 1,500 to obtain the corresponding range of the estimated minimum costs. All estimated costs ranged from 45 to 52. Because the higher the values are the longer it takes to complete a single simulation, therefore we used the values of 50 and 1,500 for “number of generations” and “population size,” respectively, as a compromise between the precision of cost estimation and the time to complete the subsequent randomizations. We used 100 randomizations to obtain the cost distribution of randomized associations. The *PACo*, version 1.1, is an *R* script and the analysis was conducted using RStudio (v. 0.99.903) (RStudio Team, 2012) running on top of *R* (v. 3.3.1) (R Core Team, 2016). We used the *cophylo* function from the *phytools* package (Revell, 2012) to visualize associations between BaMV and satBaMV isolates from China.

**FIGURE 1:**
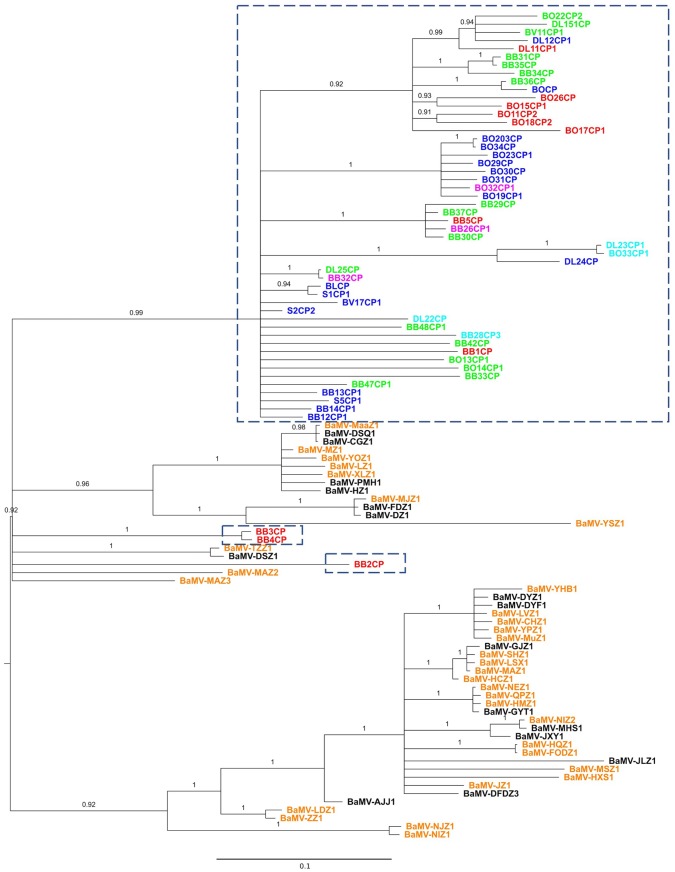
**Phylogeny of BaMV isolates from Taiwan and China.** Partial sequences of the CP gene were aligned and phylogeny reconstructed with the BI method. Isolates within the dash-lined boxes are from Taiwan; the rest, with the prefix “BaMV-,” are from China. Colored fonts indicate the general regions from which the isolates were collected: northern (blue), central (green), southern (red), northeastern (magenta), and eastern (cyan) Taiwan; southern (orange) and central (black) China. For location details, please see the **Supplementary Material S1**. The phylogeny is midpoint rooted. Numbers show the posterior probabilities (PPs). Branches with PP <0.9 are collapsed to polytomy.

**FIGURE 2:**
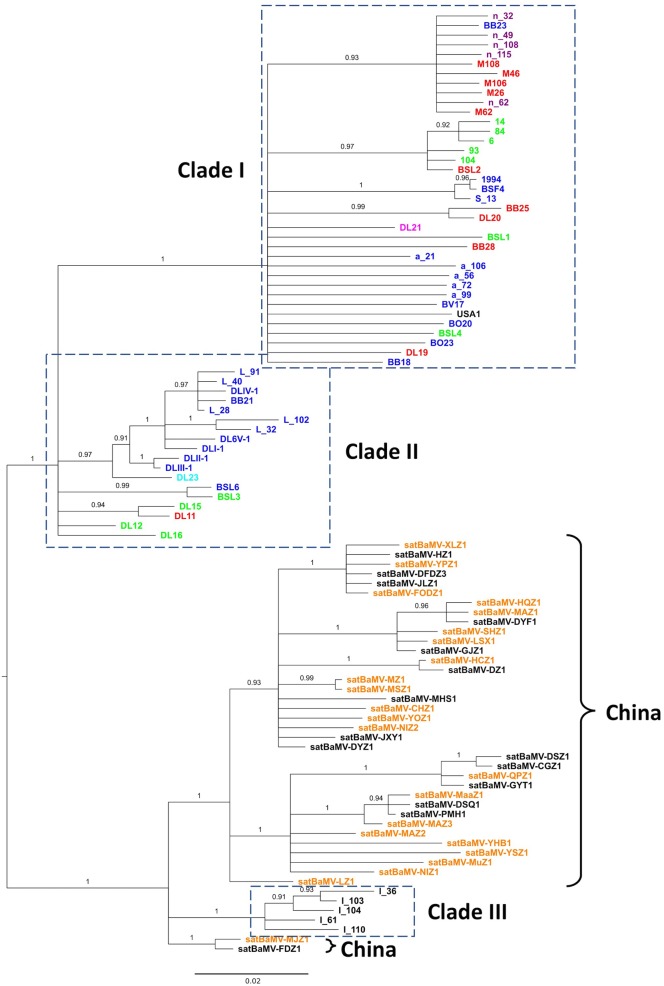
**Phylogeny of satBaMV.** Partial sequences of satBaMV isolates were aligned and phylogeny reconstructed with the BI method. Membership in previously identified three clades (Wang et al., 2014) are shown in the dash-lined boxes. Isolates from China are indicated with the right curly bracket symbols. Colored fonts, as shown in **Figure 1**, indicate the general regions from which the isolates were collected. It is to be noted that the isolates with the prefix “*n*-” in Clade I are sequences from the Hainan Island of China in our previous study. The phylogeny is midpoint-rooted. Numbers show the PPs. Branches with PP <0.9 are collapsed to polytomy.

**FIGURE 3:**
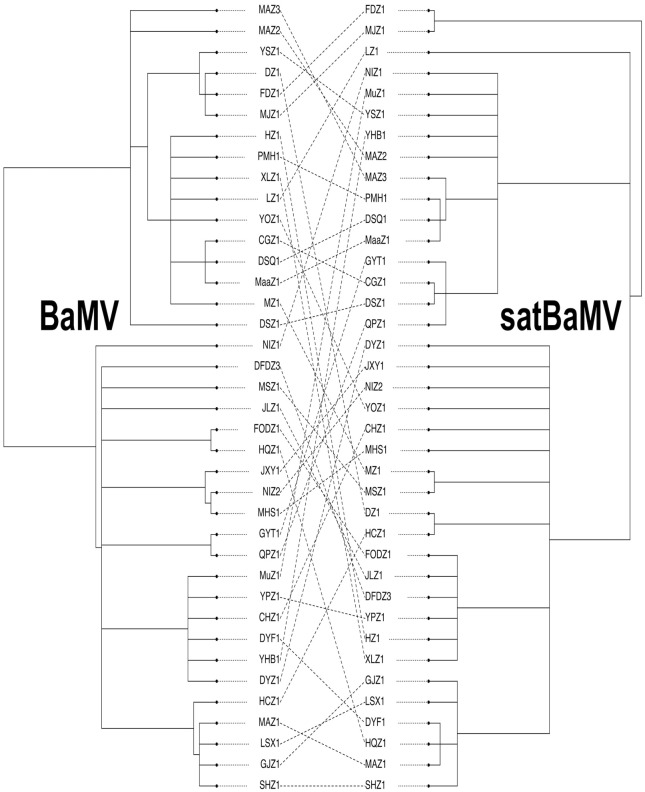
**Cophylogeny plot of BaMV and satBaMV isolates from China.** Unrooted BI trees of BaMV (left) and satBaMV (right) of 38 isolates from China were manually extracted from **Figures 1**, **2**, respectively. Isolate names are shown at the tips of each branch. Dashed lines connect BaMV and satBaMV isolates collected from the same infected bamboo host.

The input files for these two analyses are based on the collapsed Bayesian trees shown in **Figures 1**, **2**, although they are not the same. For the *PACo* analysis, which requires the information on branch length, the original tree files from **Figures 1**, **2** were used. For the *Jane* analysis, which requires each “parasite species” to have at least one corresponding “host species,” so the tree file for **Figure 2** is not suitable for the analysis. To circumvent this problem, the tree topologies of the 38 Chinese BaMV and satBaMV isolates were manually extracted from **Figures 1**, **2** to be used as the input files for the *Jane* analysis. The *cophylo* plot in **Figure 3** also used the same input file used in the *Jane* analysis to simplify the presentation.

## Results and Discussion

### Phylogeography of BaMV

Although BaMV has been detected in various parts of the world (Nelson and Borth, 2011), almost all available BaMV sequences are derived from samples obtained in Taiwan. Recent additions from China (Lin et al., 2015, 2016) provide an opportunity to explore the evolutionary history among the BaMV isolates over a larger geographic area.

A total of 101 BaMV sequences, 53 from Taiwan and 48 from China, were used to reconstruct the phylogenetic relationship. The 528-nucleotide-long sequences (nucleotides 5611–6138, with BaMV-S genome as the reference, see Materials and Methods) encompass part of the CP gene, encoding 176 of the full-length 242 amino acid residues. To establish the character polarity, e.g., place of origin, a rooted tree is required. Although there are several approaches to rooting a phylogenetic tree (Kinene et al., 2016), the most commonly used are outgroup rooting and midpoint rooting. The outgroup rooting requires a homologous sequence known *a priori*. Typically, if possible, the outgroup rooting is preferred. Alternatively, the midpoint rooting is commonly used when no suitable outgroup is available. The midpoint rooting is found to be relatively successful at identifying the root of a phylogenetic tree (Hess and Russo, 2007). In this study, we used the BI and the ML methods to infer evolutionary histories among the BaMV isolates. For each method, we also reconstructed the trees with or without the presence of an outgroup sequence to evaluate the proper placement of the root. Of the two reconstruction methods, the BI method with midpoint rooting was able to produce a reasonably resolved phylogenetic relationship with high branch supports (**Figure 1**). On the other hand, the ML method produced a star phylogeny (polytomy) encompassing the majority of the sequences, thus rendering most of the relationships unresolved (see **Supplementary Material S3**). Furthermore, the phylogenetic relationships are better resolved with midpoint rooting than with outgroup rooting. This may be due to the fact that, over the region analyzed, the outgroup FoMV CP sequence shows a relatively low sequence identity of 29.5% with that of the BaMV CP. At the nucleotide level, the average number of nucleotide difference between the FoMV CP and BaMV CP gene is 276, while the average nucleotide difference between two BaMV sequences is only 64. It is likely that some nucleotide differences among the BaMV CP sequences are seen as “noises,” thus not contributing to the resolution of their relationships (Li and Graur, 1991; Mount, 2004). Nevertheless, despite the seemingly unresolved trees, the topologies of BI and ML trees are consistent in crucial aspects that are pertinent to our study. Therefore, we will focus our discussions based on the midpoint-rooted BI tree, as shown in **Figure 1**.

Several interesting patterns emerge from **Figure 1**. First, the majority of the isolates from Taiwan form a single clade and those from China two clades. There is no single instance in which the Chinese and Taiwanese isolates are found interspersed with each other. This complete coincidence between clade formation and geographic origin suggests that BaMV has a relatively independent evolutionary history in these two geographically proximate locations. Second, one of the Chinese clades forms the basal group of the tree, suggesting that the Taiwanese isolates originated in China. More limited studies, but with full-length genomic sequences, showed the same pattern as well (Lin et al., 2016, 2017b). Third, multiple instances of independent BaMV introduction from China to Taiwan are discernible from the reconstructed phylogenetic trees.

While illuminating, these patterns are not unexpected, for the inferred direction of BaMV introduction followed the same typical route of species introduction/invasion from the mainland to an island (MacArthur and Wilson, 1967; Lomolino et al., 2010). With the frequent exchange of goods and movement of both people and agricultural practices between Taiwan and China since ancient times, multiple, independent introductions of pathogens, including viruses, should be expected. However, we note that these BaMV isolates are not contemporaneous. The isolates from Taiwan were collected between 1994 and 2000 (Yeh et al., 2004) and those from China in 2014 (see **Supplementary Material S1** for details). It is not clear whether almost two decades of difference in evolution could drastically alter the inferred tree topology, thus resulting in an erroneous inference of movement direction (e.g., the current isolates from China may actually be derived from the older isolates from Taiwan). Nevertheless, our current analysis provides motivation for the need of a more systematic and wider geographic sampling of BaMV in the field.

### Phylogeography of satBaMV

Our previous analysis on the evolution of satBaMV revealed three well-supported clades. Clade I is composed of isolates from Taiwan (collected from various bamboo species from various locations on the island, see **Supplementary Material S1** for details) and southern China (Hainan Island, specifically). Clade II is composed of isolates from the infected Ma bamboo (*D. latiflorus* Munro) in Taiwan, and Clade III of isolates from India (Wang et al., 2014). The recently available satBaMV sequences from China are collected from a much wider geographic area (Lin et al., 2015, 2017a), thus providing an opportunity to infer the phylogeography of satBaMV isolates. In this study, a total of 98 sequences were analyzed, with 60 previously analyzed and published data and 38 recently added sequences from China. The 472-nucleotide-long sequences [nucleotides 271–742, with satBaMV-BSF4 genome (AY205227) (Lin and Hsu, 1994) as the reference] encompass part of the *P20* gene, encoding 146 of the full-length 183 amino acid residues. Since the CP gene from SPMV (GenBank accession number NC_003847) is the most closely related sequence to satBaMV’s P20 gene, it is used as an outgroup to root the satBaMV tree. Over the region analyzed, the average number of nucleotide difference between SPMV CP and satBaMV sequence is 159, while the average difference between two satBaMV sequences is 23. It is apparent that the SPMV CP gene is only distantly related to the satBaMV CP gene. For this reason, we focus on midpoint-rooted BI tree for our discussion.

As shown in **Figure 2**, two interesting patterns emerge. First, the inferred midpoint-root bisects the satBaMV phylogeny at the point where it separates the previously defined Clades I and II into one of the dichotomous branches and Clade III into the other. That is, the satBaMV isolates collected from Taiwan are more closely related to each other than those collected from infected *B. vulgaris* bamboos in India (Wang et al., 2014). The same tree topology is also shown in the midpoint-rooted ML tree (see the **Supplementary Material S3**). This pattern suggests a deep genetic differentiation of satBaMV isolates between China and Taiwan. The only region where Chinese and Taiwanese isolates co-mingled is the Hainan Island of China. However, we note that the outgroup-rooted BI tree shows a different tree topology, with the root being placed within the Clade I, from which Clades II, III, and the Chinese isolates are derived (see the **Supplementary Material S3**). Such a tree topology would imply an intriguing Taiwan-origin hypothesis for the way satBaMV evolved and migrated within the Asian continent. Second, more interestingly, the isolates from China, instead of scattering throughout the satBaMV phylogeny, are clustered together and are most closely related to those from India. This pattern further accentuates our finding that geographic distance (long distance between India and China versus short distance between Taiwan and China) does not seem to be a major determinant of phylogenetic relatedness among these satBaMV isolates. The relative lack of geographic differentiation is also seen within the East Asian continent. All of Chinese isolates are from three locations: two of them (Fujian Agriculture and Forestry University and Fuzhou National Forest Park) are in the city Fuzhou, Fujian; the other is in the city Chengdu (Wangjiang Park), Sichuan. Despite a distance of approximately 1,500 km between these two cities, there is no discernible clustering of isolates based on locations. As mentioned previously, there is an overwhelming clustering of Taiwanese and Chinese isolates, despite a much shorter distance of approximately 250 km between Taipei and Fuzhou. Therefore, we hypothesize that the Taiwan Strait forms a physical barrier, thus greatly limiting gene flow between mainland China and Taiwan. However, we note that satBaMV sequences from the Hainan Island, China are clustered with those from Taiwan, despite large swaths of South China Sea in between these two islands.

Taken together with the results from BaMV, we conclude that, despite gene flow, as evidenced by several independent introductions of BaMV from China to Taiwan, both BaMV and satBaMV in Taiwan have diverged greatly from those on the Asian continent. Although we do not have BaMV sequences from India or the Hainan Island, we predict that the Indian BaMV should be found allied with those in China, while the Hainan Island BaMV should cluster with those from Taiwan. Our current study also highlights the need for a more systematic sampling in various parts of the Asian continent proper and outlying islands, especially islands of Southeast Asia, to give us a more comprehensive picture on the origin, evolution, and phylogeography of BaMV and satBaMV.

### Coevolution between BaMV and satBaMV

When a parasite depends completely on its host for vital functions, it is ordinarily anticipated that both the parasite and host will coevolve. Therefore, speciation of the host should lead to corresponding speciation of the parasite, essentially coevolution via descent. Alternatively, evolution of parasite host range can sometimes lead to colonization of a new host species phylogenetically distant from the original host, thus resulting in coevolution through colonization. The pattern of cophylogeny between the host species and the parasite species can be used to differentiate these two alternatives. Strict congruence between the host and parasite phylogenies suggests coevolution by descent. Otherwise, incongruence indicates events of shifted or expanded host range in the parasite. We reasoned that the cophylogeny study, commonly applied to investigating coevolution between host and parasite at the species level, can be used to investigate coevolution between BaMV and satBaMV at the population level as well. Of the 48 available BaMV sequences from China, 38 have corresponding satBaMV sequences that are isolated from the same infected bamboo (Lin et al., 2015, 2016, 2017a). These sequence pairs provide us an opportunity to explore the level of coevolution between the host BaMV and the parasite satBaMV.

We employed a cophylogeny plot, as shown in **Figure 3**, to visualize the degree of congruence between the BaMV and satBaMV phylogenies. **Figure 3** reveals a complex association pattern, indicating instances of incongruence between the two phylogenies (in the form of crisscrossing lines connecting individual BaMV/satBaMV pairs isolated from the same infected bamboo). We then used more quantitative approaches to explore the cophylogeny between BaMV and satBaMV from China.

Two general approaches are frequently used for cophylogeny study: global-fit and event-based methods, each has their advantages and disadvantages (Desdevises, 2007; Filipiak et al., 2016). In this study, we used *PACo* (Procrustean Approach to Cophylogeny) (Balbuena et al., 2013), a global-fit method, and *Jane* (Conow et al., 2010), an event-based method, to investigate the pattern of cophylogeny between BaMV and satBaMV sequences. An overall congruence would suggest that the BaMV/satBaMV pair in an infected host is frequently co-transmitted to a new host bamboo in their evolutionary history. In contrast, an incongruence between BaMV and satBaMV phylogenies would suggest relatively independent transmission histories of these two entities. Trees presented in **Figures 1**, **2** are used as the data for each analysis.

Procrustean Approach to Cophylogeny uses the statistical process of Procrustes superimposition to obtain the Procrustes distance between two objects as a measure of “similarity in shape” (called “global goodness-of-fit,” symbolized by *m*^2^_XY_). In the context of the cophylogeny analysis, the objects are the topologies of the host tree and the parasite tree. The significance of the observed *m*^2^_XY_ can then be assessed by comparing to the distribution of sample *m*^2^_XY_ generated through randomized associations between the host and parasite taxa. With the current topologies of the BaMV and satBaMV trees, the observed *m*^2^_XY_ is 1.715, while the mean *m*^2^_XY_ for the random samples is 2.047 (obtained from 100,000 randomizations). Since the resulting probability is 1.71 × 10^-3^, we can reject the null hypothesis that the topology of the BaMV (host) tree cannot predict the topology of the satBaMV (parasite) tree. That is, at least a significant portion of the satBaMV tree topology depends on (i.e., can be predicted by) the BaMV tree topology. Such a dependency suggests the presence of coevolution.

The advantage of the global-fit method, such as *PACo*, is that we can quickly obtain a statistical fit between two phylogenies without intensive computation. Its disadvantage is that we cannot have a detailed sense of what may have been the evolutionary events responsible for the observed pattern. For the event-based method of *Jane* (Conow et al., 2010), five mutually exclusive and exhaustive event types – cospeciation, duplication, duplication and host switch, loss, and failure to diverge – are assumed responsible for any given cophylogeny pattern. Various costs, or ranges of costs, are assigned to each event *a priori*. In the default setting, only the cospeciation event does not carry a cost, all the others have varying degrees of cost associated with them. Possible historical events (solutions) were found by minimizing the total costs. The minimum cost for the hypothesized historical event(s) can then be compared to a prescribed number of sample costs obtained by finding the minimum cost for each of the randomized association between the host and parasites. The details for the chosen parameters used in the analysis can be found in Section “Materials and Methods.” In the current cophylogeny study for BaMV and satBaMV, the best minimum cost for the current trees is 48, while the sample costs from 100 randomized associations ranged from 48 to 62, with a mean of 55.65 and a standard deviation of 3.15. This result showed that the observed BaMV and satBaMV topologies have a significantly lower cost than two randomly generated trees (*p* = 3.19 × 10^-8^), suggesting that cospeciation is the major cause for the observed tree topologies. That is, BaMV and satBaMV isolates in general coevolve within the same infected bamboo individual.

Besides giving an overall statistical test, events that best fit our current cophylogeny pattern were also identified by the *Jane* analysis. Of the five possible event types, 16 “cospeciation,” 21 “duplication and host switch,” and 6 “loss” events were hypothesized. That is, a total of 43 evolutionary steps are needed to render the BaMV and satBaMV trees congruent. How do we interpret these hypothetical events in the ecological context of BaMV/satBaMV? It is interesting to note that, unlike other potexviruses, it is somewhat difficult to infect bamboos with BaMV/satBaMV preparation, even in the laboratory setting. Since bamboos are perennials, with some species having a lifespan of up to several decades, the difficulty in BaMV/satBaMV transmission would suggest a state of chronic infection. That is, the within-host population dynamics of viral infection is likely dominated by a set of BaMV/satBaMV that, given time, will coevolve from, say, the ancestral A/a sequences (or closely related mutant swarms) to the descendant A_1_/a_1_. If the ancestral set was also able to infect and establish in a different host successfully, the set can also coevolve to A_2_/a_2_ via accumulation of phylogenetically informative mutations either independently, like genetic drift, or driven by natural selection, e.g., in the form of evolutionary arms race. That is, the typical ecological process of infection, in the context of BaMV/satBaMV biology, can be seen as the source of “cospeciation” event, as defined by the *Jane* analysis. If the A/a set infects a bamboo that is already infected with a phylogenetically more distant coevolving set, say C/c, the A/a set can completely take over the C/c sequences, thus resulting in a cospeciation event. Or the process of superinfection can result in the successful establishment of A/c or C/a pairing, which, given time, will coevolve to A_3_/c_3_ or C_3_/a_3_, respectively. Either way, a “loss” and a “duplication and host switch” event will then be counted in the *Jane* analysis.

Despite the apparent difficulty in transmitting BaMV/satBaMV from individual to individual bamboo, our cophylogeny analyses showed that successful establishment of infection or differential survival of superinfection by phylogenetically distinct BaMV and/or satBaMV sequences may not be uncommon. In fact, an *in planta* experiment showed differential accumulations of viral progeny between two BaMV isolates, suggesting one isolate having a fitness advantage over the other during coinfection, and presumably superinfection as well (Lin et al., 2017b). However, it is to be noted that all the Chinese isolate used in the cophylogeny analysis were collected in three areas and from many different bamboo species. All the sampling sites are in a botanical garden setting (W. Lin, personal communication), therefore, all infected bamboo hosts are presumed to be in close proximity to each other. However, we did not observe obvious clustering of sequences based on location of sampling (see **Figure 2**), suggesting that at least some of the BaMV/satBaMV isolates were preexisting before being placed in the same botanical gardens. Unfortunately, the actual geographic origins and natural host species are not clear for these BaMV/satBaMV isolates. Again, for a more detailed phylogeographic and cophylogenetic analyses, a more systematic collection of BaMV and satBaMV isolates in the field and full genomic sequences are needed.

## Author Contributions

I-NW performed analyses and wrote the draft of the manuscript. W-BY conducted sequencing and submitted sequence information to GenBank. N-SL provided reagents and materials. All three edited the manuscript.

## Conflict of Interest Statement

The authors declare that the research was conducted in the absence of any commercial or financial relationships that could be construed as a potential conflict of interest.

## Footnotes

1 http://tcoffee.crg.cat/apps/tcoffee/do:expresso

2 http://www.ebi.ac.uk/Tools/msa/clustalo/

3 http://www.atgc-montpellier.fr/phyml/

## Supplementary Material

**SUPPLEMENTARY MATERIAL S1 | BaMV and satBaMV isolates information.**

**SUPPLEMENTARY MATERIAL S2 | Map of BaMV and satBaMV sampling locations.**

**SUPPLEMENTARY MATERIAL S3 |** **(1)** Bayesian BaMV CP gene tree with FoMV CP gene as the outgroup. Numbers show the posterior probabilities (PP). Branches with PP < 0.9 are collapsed to polytomy. **(2)** Maximum likelihood BaMV CP gene tree with midpoint rooting. Numbers show the bootstrap values (out of 100). Branches with bootstrap values < 375 (out of 500) are collapsed to polytomy. **(3)** Maximum likelihood BaMV CP gene tree with FoMV CP gene as the outgroup. Numbers show the bootstrap values (out of 500). Branches with bootstrap values < 375 are collapsed to polytomy. **(4)** Bayesian satBaMV tree with SPMV CP gene as the outgroup. Branches with posterior probability < 0.9 are collapsed to polytomy. **(5)** Maximum likelihood satBaMV tree with midpoint rooting. Branches with bootstrap values < 375 (out of 500) are collapsed to polytomy. **(6)** Maximum likelihood satBaMV tree with SPMV CP gene as the outgroup. Branches with bootstrap values < 375 (out of 500) are collapsed to polytomy.
