# Supplementary figures and images for: Phylogeography and Coevolution of Bamboo Mosaic Virus and Its Associated Satellite RNA
Source: Front Microbiol. 2017 May 23;8:886. doi: 10.3389/fmicb.2017.00886 (PMC5440514; doi:10.3389/fmicb.2017.00886)

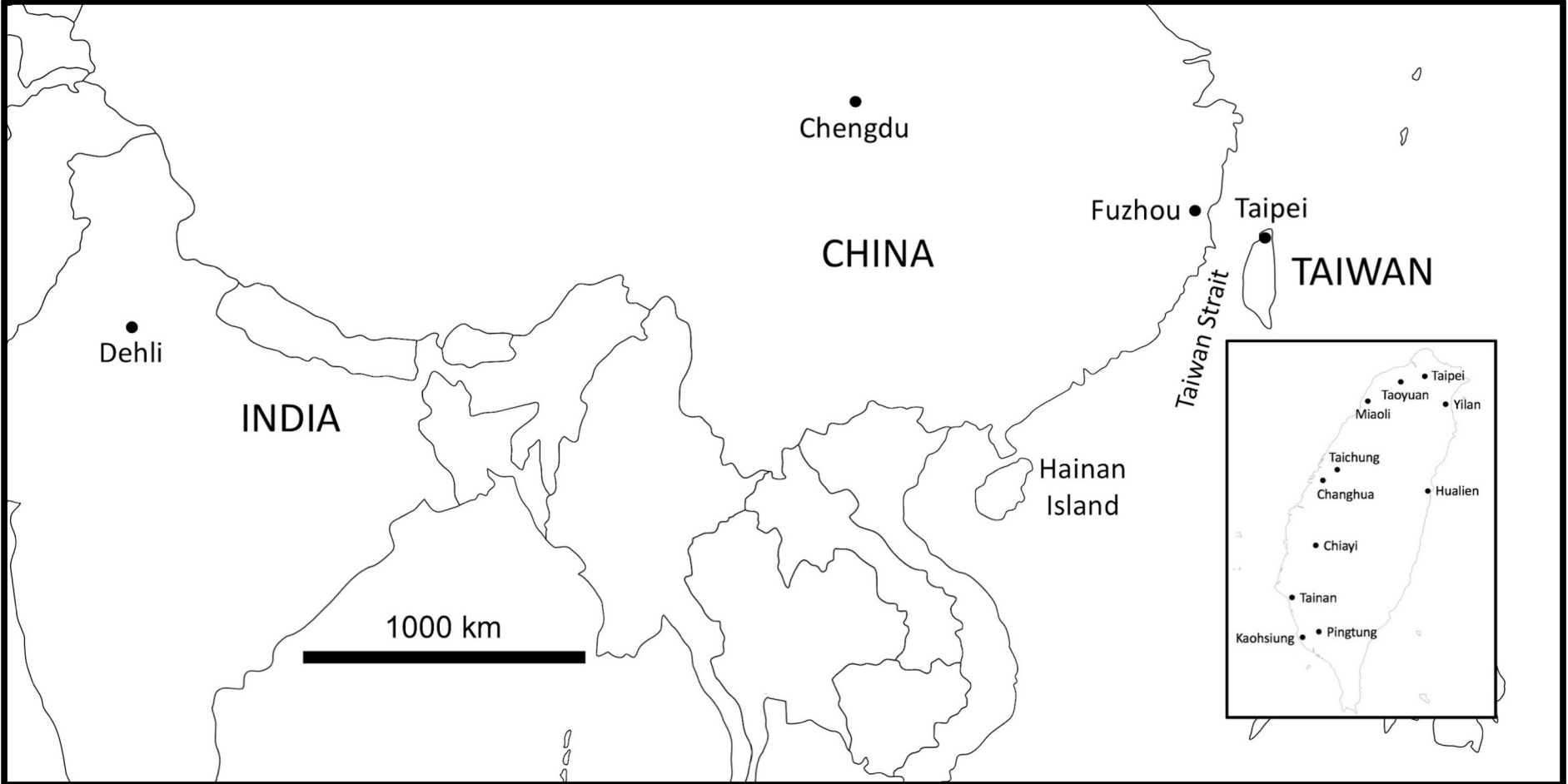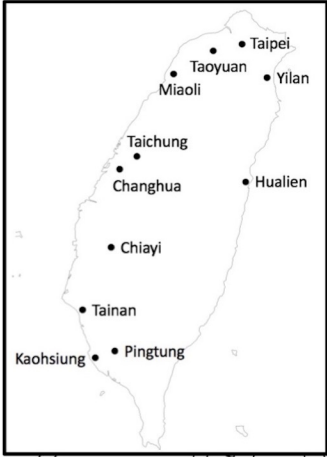

Supplement: Supplementary file 2 [file Data_Sheet_2.pdf]
