# Supplementary material for: Phylogeography and Coevolution of Bamboo Mosaic Virus and Its Associated Satellite RNA
Source: Front Microbiol. 2017 May 23;8:886. doi: 10.3389/fmicb.2017.00886 (PMC5440514; doi:10.3389/fmicb.2017.00886)

Supplementary Material S3-1. Bayesian BaMV CP gene tree with FoMV CP gene as the outgroup. Numbers show the posterior probabilities (PP). Branches with PP < 0.9 are collapsed to polytomy.

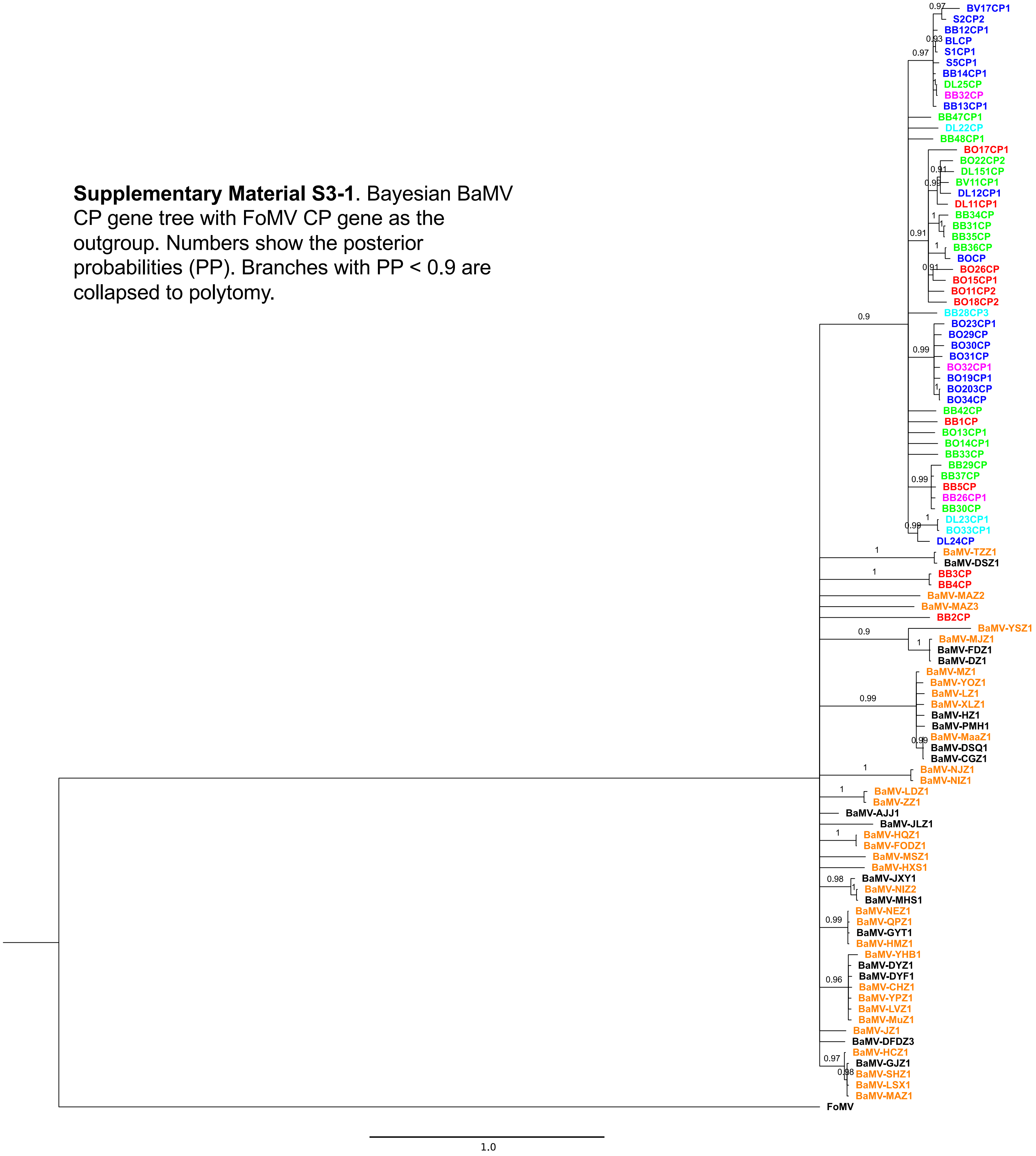

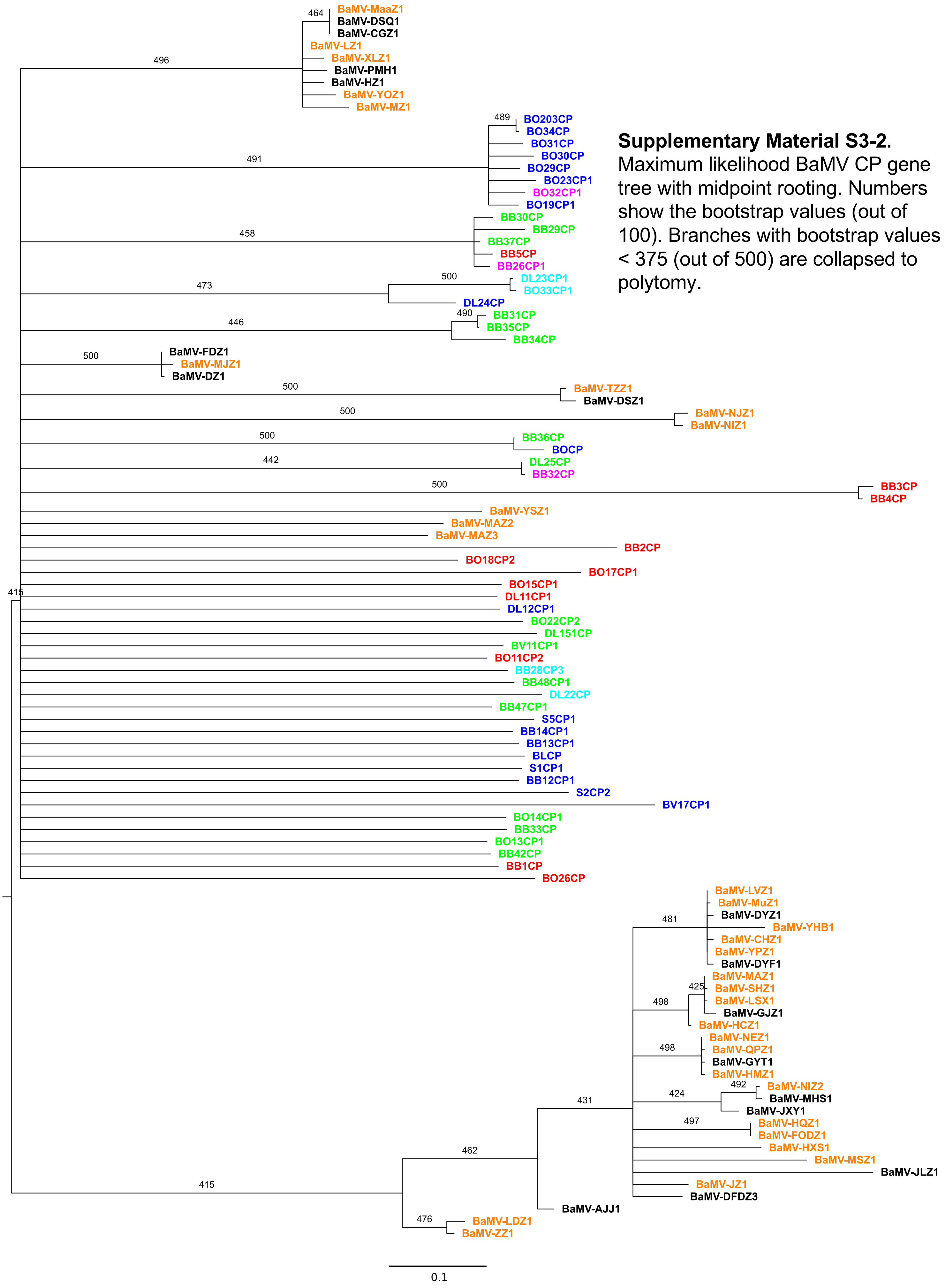

Supplementary Material S3-2. Maximum likelihood BaMV CP gene tree with midpoint rooting. Numbers show the bootstrap values (out of 100). Branches with bootstrap values < 375 (out of 500) are collapsed to polytomy.

Supplementary Material S3-3. Maximum likelihood BaMV CP gene tree with FoMV CP gene as the outgroup. Numbers show the bootstrap values (out of 500). Branches with bootstrap values < 375 are collapsed to polytomy.

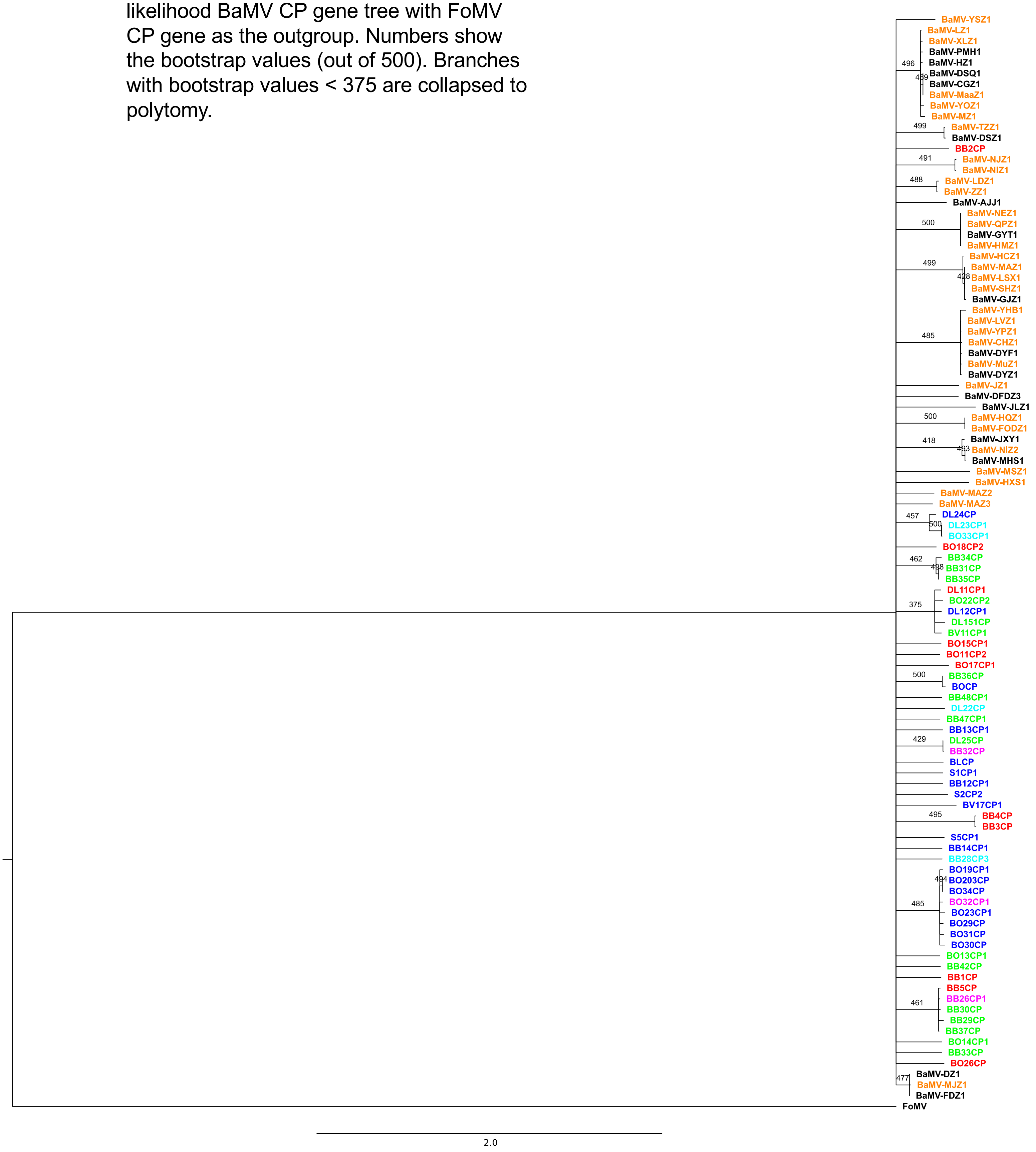

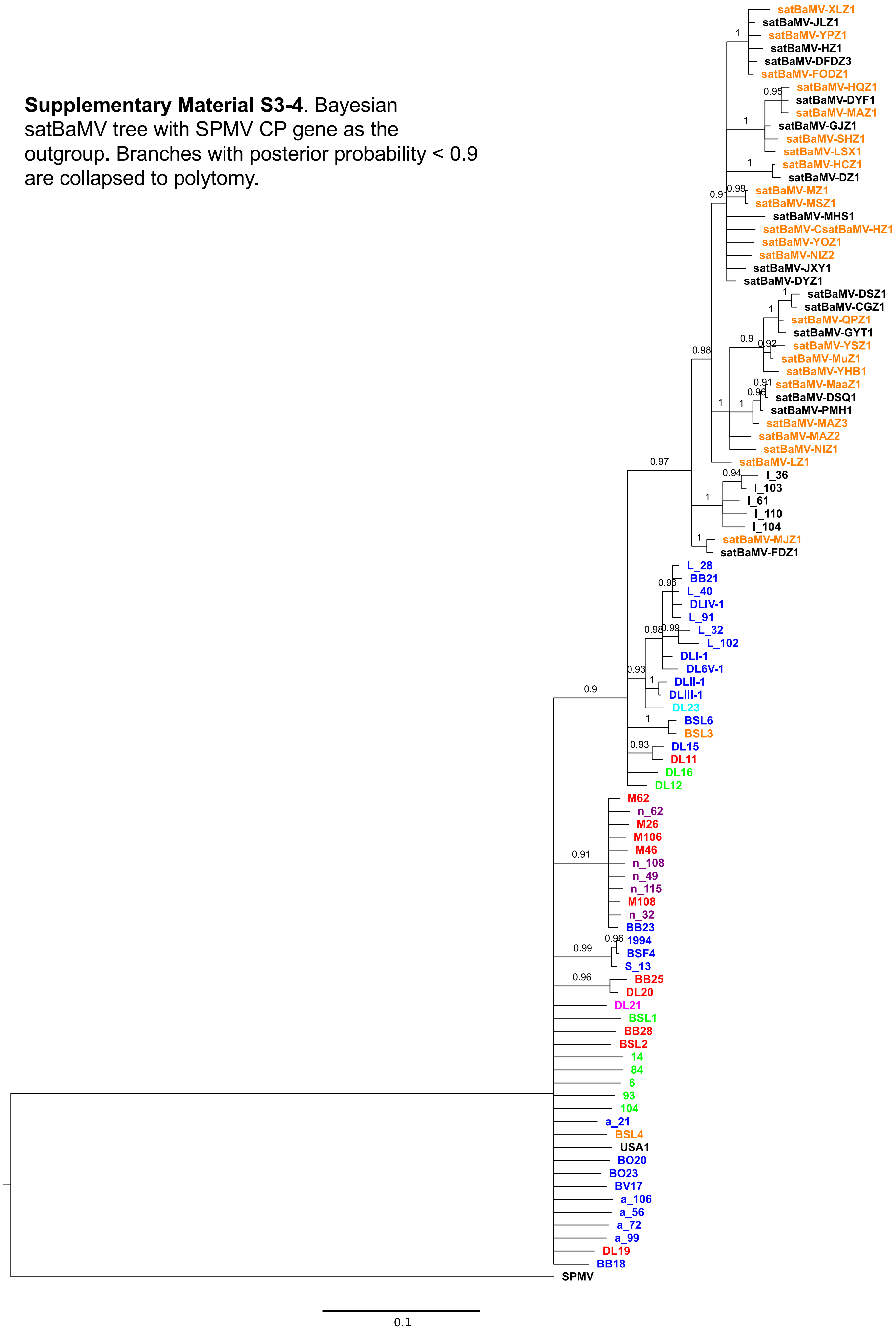

Supplementary Material S3-4. Bayesian satBaMV tree with SPMV CP gene as the outgroup. Branches with posterior probability < 0.9 are collapsed to polytomy.

Supplementary Material S3-5. Maximum likelihood satBaMV tree with midpoint rooting. Branches with bootstrap values < 375 (out of 500) are collapsed to polytomy.

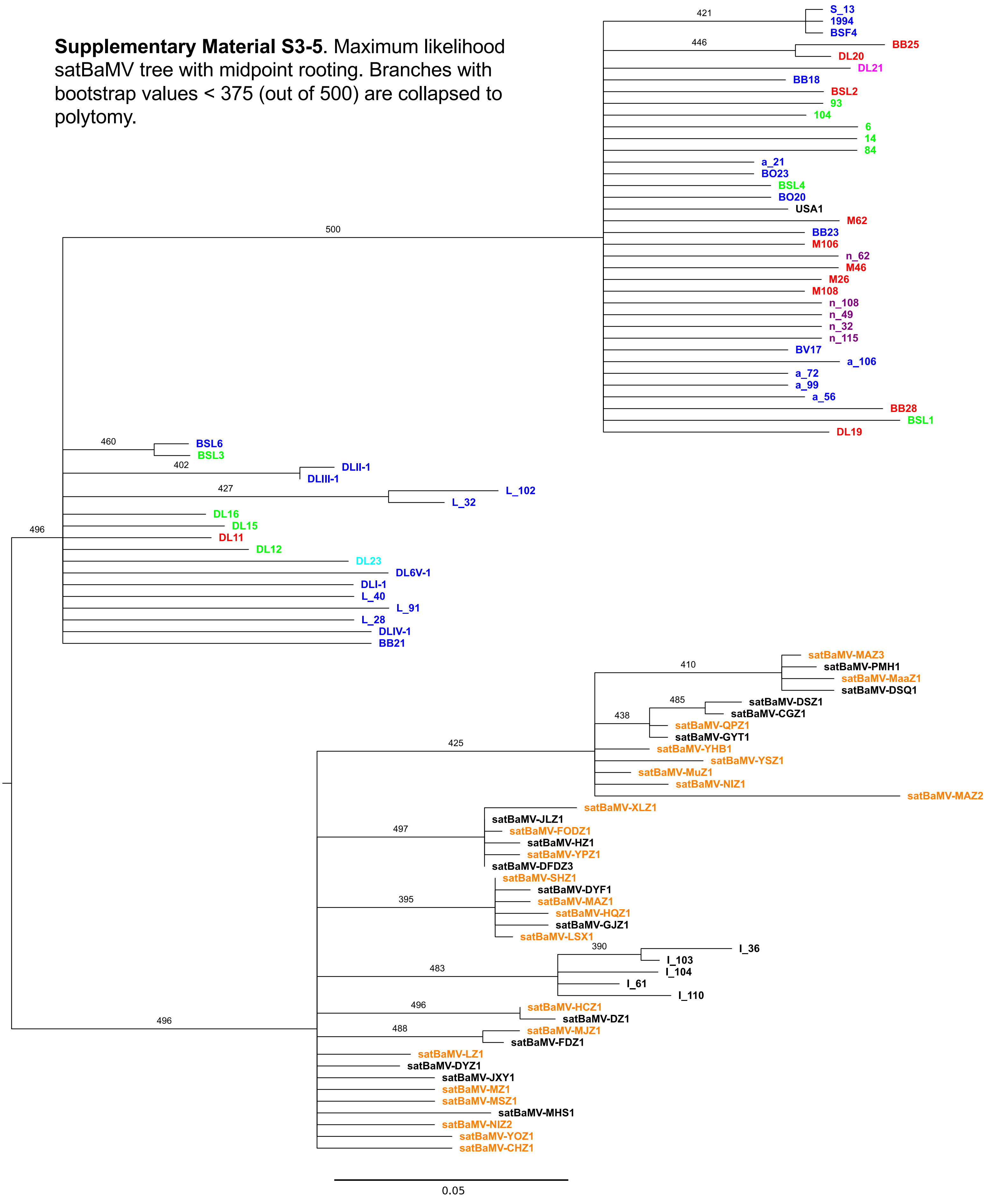

Supplementary Material S3-6. Maximum likelihood satBaMV tree with SPMV CP gene as the outgroup. Branches with bootstrap values < 375 (out of 500) are collapsed to polytomy.

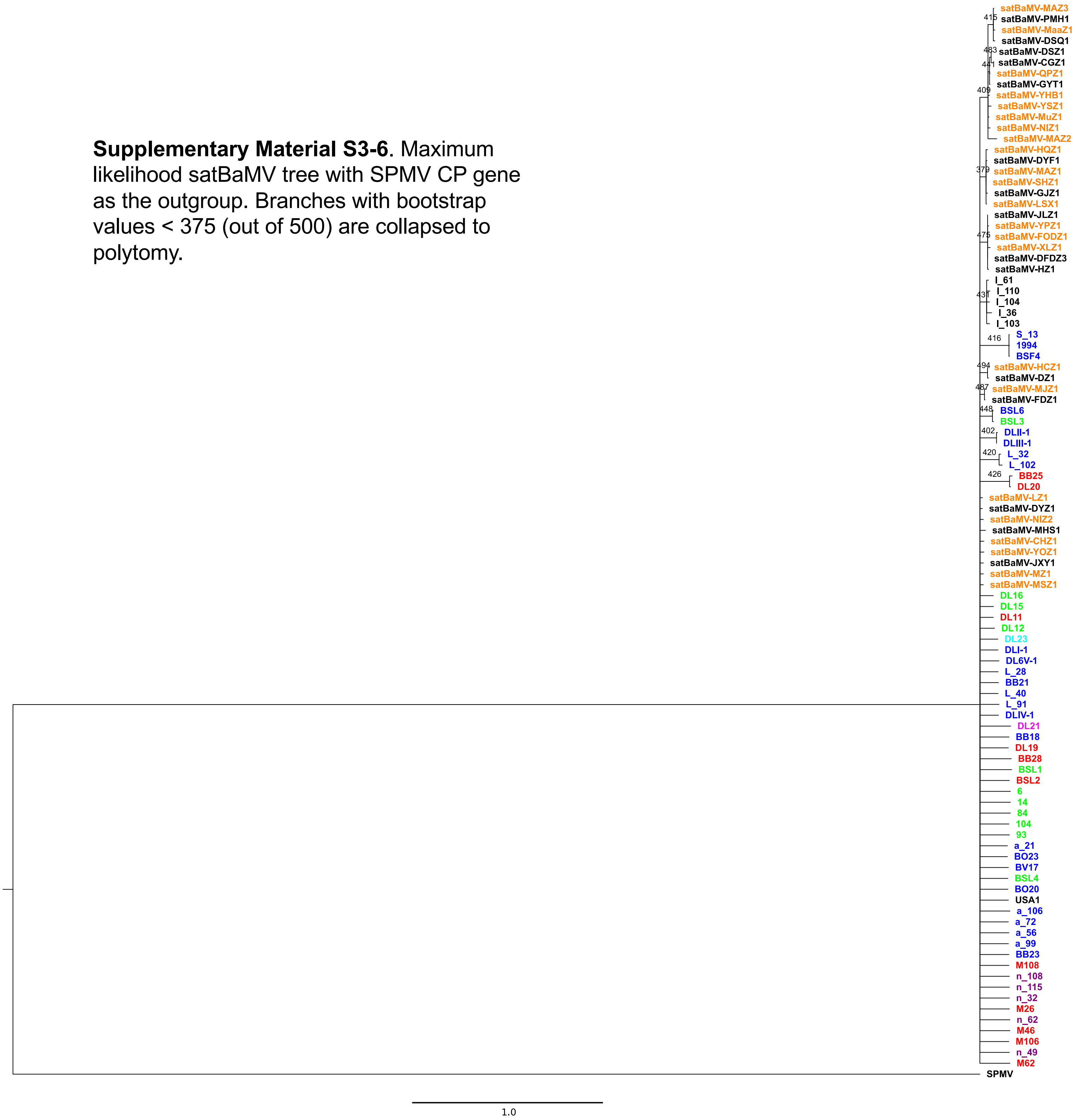

Supplement: Supplementary file 3 [file Data_Sheet_3.pdf]
